# D-dimer for risk stratification and antithrombotic treatment management in acute coronary syndrome patients: a systematic review and metanalysis

**DOI:** 10.1186/s12959-021-00354-y

**Published:** 2021-12-18

**Authors:** Flavio Giuseppe Biccirè, Alessio Farcomeni, Carlo Gaudio, Pasquale Pignatelli, Gaetano Tanzilli, Daniele Pastori

**Affiliations:** 1 Department of Clinical Internal, Anesthesiological, and Cardiovascular Sciences, Sapienza University of Rome, Viale del Policlinico 155, 00161 Rome, Italy; 2 Department of General and Specialized Surgery “Paride Stefanini”, Sapienza University of Rome, Viale del Policlinico 155, 00161 Rome, Italy; 3 Department of Economics and Finance, University of Rome “Tor Vergata”, Via Columbia 2, 00133 Rome, Italy

**Keywords:** D-dimer, Myocardial infarction, No-reflow phenomenon, Prognosis, Acute coronary syndrome

## Abstract

**Background:**

Data on the prognostic role of D-dimer in patients with acute coronary syndrome (ACS) are controversial. Our aim was to summarize current evidence on the association between D-dimer levels and short/long-term poor prognosis of ACS patients. We also investigated the association between D-dimer and no-reflow phenomenon.

**Methods:**

Systematic review and metanalysis of observational studies including ACS patients and reporting data on D-dimer levels. PubMed and SCOPUS databases were searched. Data were combined with hazard ratio (HR) and metanalysed. The principal endpoint was a composite of cardiovascular events (CVEs) including myocardial infarction, all-cause and cardiovascular mortality.

**Results:**

Overall, 32 studies included in the systematic review with 28,869 patients. Of them, 6 studies investigated in-hospital and 26 studies long-term outcomes. Overall, 23 studies showed positive association of high D-dimer levels with CVEs. D-dimer levels predicted poor prognosis in all studies reporting in-hospital outcomes. Five studies satisfied inclusion criteria and were included in the metanalysis, with a total of 8616 patients. Median follow-up was 13.2 months with 626 CVEs. The pooled HR for D-dimer levels and CVEs was 1.264 (95% CI 1.134–1.409). Five out of 7 studies (4195 STEMI patients) investigating the association between D-dimer levels and no-reflow showed a positive correlation of D-dimer levels with no-reflow.

**Conclusions:**

In patients with ACS, D-dimer was associated with higher in-hospital and short/long-term complications. D-dimer was also higher in patients with no-reflow phenomenon. The use of D-dimer may help to identify patients with residual thrombotic risk after ACS.

**Trial registration:**

The review protocol was registered in PROSPERO International Prospective Register of Systematic Reviews: CRD42021267233.

**Supplementary Information:**

The online version contains supplementary material available at 10.1186/s12959-021-00354-y.

## Introduction

Coronary artery disease (CAD) is the leading cause of death worldwide with acute myocardial infarction (MI) representing the clinical condition associated with the greatest morbidity and mortality, up to 30% of in-hospital deaths [1, 2]. Prompt reperfusion with percutaneous coronary intervention (PCI), advances in acute cardiovascular care and more effective medical therapy have improved the prognosis of patients with MI in the last years [3].

This increased survival revealed a previously unrecognized long-term risk of recurrent thrombotic events despite optimal medical therapy [2]. Indeed, almost one fifth of MI patients suffer from re-hospitalization within 1 year and 10% from recurrent MI [3, 4].

There is therefore a growing need of a more accurate risk stratification of MI patients to identify those at higher risk for adverse events and worse prognosis [5].

Current guidelines of the American Heart Association and the European Society of Cardiology highlighted the lack of useful biomarkers to predict in-hospital complications and short/long term prognosis of patients with MI, recommending for this purpose only to measure troponin and brain natriuretic peptide (BNP) serum levels, with the latter associated with a low level of evidence [6, 7].

During last decades, several studies, mostly conducted in the 90’s before the routine use of dual antiplatelet therapy, described the pivotal and prognostic role of coronary thrombosis and hypercoagulable state in the pathophysiology of MI, correlating increased coagulation activity with recurrent event and poor outcome after MI [8–10].

As a marker of rapid fibrin turnover and high thrombotic activation in both arterial and venous system, interest in D-dimer has grown over time, and its predictive role has been investigated in several acute and chronic cardiovascular care, [11]. In patients with MI, high circulating D-dimer levels have been correlated with recurrent MI and poor prognosis [12, 13] and early reduction in plasma concentration of D-dimer by Ximelagatran administration was associated with decreased risk of new cardiovascular events [14].

In addition, there are some data indicating that D-dimer may be higher in MI patients experiencing no-reflow phenomenon [15], a severe condition in which high thrombotic burden interfere with complete restoration of myocardial blood supply after PCI, known to be correlated with adverse outcomes and poor prognosis [16].

Despite many studies published, conclusive data on the real usefulness and additional value of D-dimer in the management of patients with MI are lacking.

The aim of this systematic review and meta-analysis is to summarize current evidence on the potential prognostic role of D-dimer levels in CAD patients and its association with no-reflow phenomenon.

## Materials and methods

### Strategy search

We conducted a systematic review of literature searching MEDLINE via PubMed and Scopus databases using a combination of the following keywords: “D-dimer” and “myocardial infarction” and “coronary artery disease” and “acute coronary syndrome”. There was no time restriction and the last search was run on May 1, 2021. We included only journal articles in English language with full text available. We excluded case-control studies, case reports, editorials/comments, letters, review and meta-analysis, and experimental studies. Supplementary Fig. 1 report strategy searches which were performed according to the PRISMA guidelines. The systematic review was registered at PROSPERO (https://www.crd.york.ac.uk/PROSPERO) with registration number CRD42021267233.

### Study selection and quality assessment

Two physicians (FGB, DP) independently screened the titles and abstracts of manuscripts identified through the database searches to identify studies potentially eligible for further assessment. A third physician (GT) reviewed eligible studies for appropriateness and completeness. The study selection was performed in multiple phases. In the first phase, potentially relevant studies were obtained by combined searches of electronic databases using the selected above-mentioned keywords and exclusion criteria. In the second phase, potentially eligible studies were reviewed to assess the appropriateness with the study question. Finally, the quality of pertinent studies included in the metanalysis was assessed by the Newcastle–Ottawa scale. Studies with a score ≥ 7 were considered of good quality. Quality assessment is reported in Supplementary Table 1.

### Endpoints

The principal endpoint was a composite of cardiovascular events (CVEs) including MI, all-cause and cardiovascular mortality. The type of endpoint reported in each study is given in Table 1. Given the lack of high-quality studies, the association between D-dimer and no-reflow was not formally analysed but only systematically reviewed. Ethical approval was not required given the study type.

**Table 1:** Clinical characteristics of studies investigating major adverse cardiac events and all-cause mortality included in the systematic review

Author/ year	Setting	N	Age (years)	Women (%)	Study Design	FU (months)	Endpoints	D-dimer	Main findings
**STEMI**									
Biccirè 2021 [17]	STEMI	132	64	19.1	R	In-hospital	Adverse events (cardiogenic shock, resuscitated cardiac arrest and death).	Events vs control group: log-transformed D-dimer 6.8 ± 1.1 vs 6.3 ± 0.8, *p* = 0.019	Patients experiencing in-hospital adverse events had higher values of D-dimer compared to those free from events.
Huang 2020 [18]	STEMI	1165	63.5	17	R	In-hospital	CVEs including cardiac death, non-fatal AMI, revascularization, and stroke.	≥ 800 vs < 800 ng/mL	Increased D-dimer level predicted CVEs (OR 8.408, 95% CI 4.065–17.392, *P* = 0.001). D-dimer AUC was 0.840 (95% CI 0.769–0.911). The best cut-off value was 640 ng/mL. In the subgroup with no-reflow phenomenon, increased D-dimer predicted CVEs (OR 8.114, 95%CI 1.598–41.196, *p* = 0.012)
Luo 2020 [19]	STEMI	400	62.5	21	R	12	CVEs (all-cause death, TVR, MI, UA, HF, stroke or TIA)	Groups (μg/L) 1: 74.0; 2: 146.0; 3: 256.5; 4: 576.0.	The incidence of CVEs and all-cause mortality within 30 days (*p* < 0.001), 6 months (*p* = 0.001), and 1 year (p = 0.001) after PCI in the highest quartile of the D-dimer groups were higher than those in the other 3 groups.
Qi Zhou 2020 [20]	STEMI	872	63.7	19.8	R	29	All-cause mortality	Groups (μg/mL) 1: ≤0.33; 2: 0.33–0.64; 3: 0.64–1.33; 4: ≥1.33.	Higher in-hospital HF (40.2 vs 10.2%, *p* < 0.0001), malignant arrhythmia (14.2 vs 2.3%, *p* < 0.0001), and all-cause mortality (5.9 vs 0%, p < 0.0001) rates were observed in Group 4. 84 patients died. Group 4 was a predictor of all-cause mortality (HR: 2.53, 95%CI 1.02–6.26, *p* = 0.045).
Lin 2020 [21]	STEMI	550	63.5	12.2	P	16	CI-AKI, in-hospital outcomes and long-term mortality and CVEs §	D-dimer quartiles (μg/mL): 1: < 0.38; 2: 0.38–0.67; 3: 0.68–1.03; 4: > 1.03 .	D-dimer > 0.69 μg/mL was an independent risk factor for long-term mortality (HR: 3.41 [95% CI, 1.4–8.03], *p* = 0.005) and CVEs
Zhang 2018 [22]	STEMI	926	52.6	54.7	P	In-hospital	Mortality	383.1 ng/mL ± 264.2	Patients without pre-infarction angina with high D-dimer level on admission had significantly increased in-hospital mortality compared to the other patients (*p* = 0.041).
Gao 2018 [23]	STEMI with T2DM	822	62.5	46.1	P	100	Mortality	D-dimer 430.0 ng/mL ± 256.8	Patients with high plasma D-dimer level on admission showed a significantly shorter survival time (*p* < 0.001 in the log-rank test).
Hansen 2018 [24]	STEMI	971	61	20	cross-sectional cohort study	55	Composite of all-cause mortality, reinfarction, stroke, unscheduled revascularization, HF rehospitalization Secondary outcome was total mortality	Median D-dimer 456 ng/mL (IQR 286–801).	Adjusted OR for composite endpoints for D-dimer above 456 ng/mL: 1.179 (95% CI, 0.814–1.706 *p* = 0.384) Adjusted OR for total mortality for D-dimer above 456 ng/mL: 2.01 (95% CI, 1.06–3.83; *p* = 0.034)
Sarli 2015 [25]	STEMI	266	64	38	P	in-hospital	CVEs: nonfatal MI, in-stent thrombosis, and in-hospital mortality during hospitalization.	D-dimer (μg/l): 686 (±236) vs 418 (±164), *p* < 0.001.	D-dimer level predicted CVEs (OR: 1.002; 95% CI: 1.000–1.004; *p* = 0.029). Optimal cut-off value was 544 μg/mL for CVEs.
Erkol 2014 [15]	STEMI	569	56	16	A	38	Mortality and CVEs (death, non-fatal MI, stroke, revascularization, and advanced HF at long-term follow-up)	D-dimer overall 0.40 mg/L (0.20–0.87).	Univariable HR for long-term mortality 1.56 (95%CI, 1.24–1.95, p < 0.001) and CVEs 1.60 (95%CI, 1.37–1.83, *p* < 0.001); not significant association at multivariable analysis.
HORIZONS-AMI substudy 2014 [26]	STEMI	461	1st: 55.8 2nd: 61.0 3rd: 70.2	20.61	R	36	CVEs (composite of all-cause death, recurrent MI, stroke, or TVR for ischemia.)	Tertiles (μg/mL): 1: < 0.30 (*n* = 215) 2: 0.30–0.71 (*n* = 161) 3: ≥0.71 (*n* = 85)	D-dimer levels ≥0.71 μg/mL on admission predicted CVEs (HR 2.58 [95% CI, 1.44–4.63], *p* = 0.0014), compared to the lowest group.
Ozgur Akgul 2013 [27]	STEMI	453	55.6	19.65	P	6	Mortality	High D-dimer group: > 0.72 μg/mL; Low D-dimer group: lowest two tertiles (≤0.72 μg/mL).	Highest tertile of D-dimer associated with in-hospital CV mortality and 6-month all-cause mortality (7.2 vs. 0.6%, *p* < 0.001 and 13.9 vs. 2%, p < 0.001, respectively). Fatal reinfarction, advanced HF, and CVEs were more frequent in high D-dimer group (p < 0.001).
Pineda 2010 [28]	AMI STEMI (85.9%)	142	41	8.45	P	36	Adverse CV events included stroke, ACS, CABG/PCI, hospitalisation due to congestive HF, CV and global mortality.	Event 360.0 ng/mL vs no event 297.5 ng/mL, *p* = 0.314	No significant differences in D-dimer levels in the event group.
**NSTEMI**									
Lu 2021 [29]	NSTEMI	1357	65	31.4	P	12	Mortality and CVEs including all-cause death, hospital admission for UA and/or HF, nonfatal recurrent MI and stroke)	0.380 μg/mL (0.27–0.65) Event group (mortality at 1 year): 0.95 (0.50, 2.00) Control group: 0.37 (0.26, 0.60)	HR for D-dimer for 1-year death and CVEs: 2.12 (95%CI, 1.50–2.99, p < 0.0001).
Hulusi Satilmisoglu 2017 [30]	NSTEMI	234	57.2	24.8	R	14	Mortality	Non-survivors: 1568 ± 1489 ng/mL Survivors: 632 ± 995 ng/mL	D-dimer correlated with GRACE (r = 0.215, *p* = 0.01) and TIMI scores (r = 0.253, p < 0.001). Higher levels recorded for D-dimer assay (*p* = 0.003) in non-survivors. At multivariate analysis, D-dimer assay no significant predictor of increased mortality risk.
Tello-Montoliu 2007 [31]	NSTEMI	358	67.4	35.8	R	6	Death, new ACS, revascularization, and HF	Overall D-dimer level: 340 (211–615) ng/mL	Admission D-dimer levels did not predict events [HR: 1.26 (0.79–2.02), *p* = 0.337).
**AMI**									
Fu 2020 [32]	AMI with ESRD	113	69.2	33.6	R	In-hospital	Mortality	Mortality: 3.2 mg/L Survival: 1.1 mg/L *p* = 0.023	D-dimer ≥2.4 mg/L predicted in-hospital mortality (OR 2.771 [95% CI, 1.017–8.947], p < 0.001).
Wang 2020 [33]	AMI	197	Male 61.8 Female 74.2	20	P	6	All-cause mortality (in- and out-of-hospital deaths) or readmission.	Male D-dimer (mg/L) 0.4 vs 1.0 *P* < 0.001 Female D-dimer (mg/L) 0.4 vs 0.6 *p* = 0.015.	HR for continuous D-dimer in women 2.029 (95%CI, 1.403–2.933; p < 0.001). D-dimer ≥0.43 mg/L as an independent predictor of poor prognosis in female AMI patients.
Zhang 2020 [34]	AMI	4495	62	31.03	P	24	All-cause mortality	< 145 ng/mL ≥145 ng/mL	Elevated D-dimer was associated with mortality (univariable HR 1.20, 95%CI, 1.04–1.37, p = 0.01) and in the patients in different groups (HFpEF, HFrEF, non-HF).
Yu 2019 [35]	AMI	5923	62.2	30.5	P	In-hospital	Mortality	D-dimer tertiles (ng/mL): Low: ≤88; Intermediate: 89–179; High: > 179.	After multivariable adjustment, D-dimer significantly predicted in-hospital mortality (OR 1.060 [95% CI, 1.026–1.094], *p* < 0.001). D-dimer levels significantly improved the prognostic performance of GRACE score (C-statistic: *p* = 2.269, *p* = 0.023; IDI: 0.016, *p* = 0.032; no-reflowI: 0.291, *p* = 0.035).
REBUS study 2017 [36]	AMI	412	67	22.3	P	24	Composite endpoint (all-cause death, MI, congestive HF, or all-cause stroke)	Median D-dimer was 677 μg/L at inclusion	D-dimer was not associated with the composite endpoint (HR 1.22 [95% CI 0.99–1.51], *p* = 0.06, for one SD increase).
Smid 2011 [37]	AMI	135	61	26	P	12	CV death, recurrent MI, a second PCI or CABG and ischemic stroke.	On admission D-dimer: 370 (260–718) ng/mL	D-dimer on admission was higher in patients with recurrent thrombotic CV event (medians 550 vs. 365 ng/mL, p = 0.06) OR for D-dimer against endpoints: 2.9 (95%CI 0.9–8.8)
THROMBO study 2000 [38]	AMI	1045	Male 58 Female 62	24.3	P	26	Recurrent cardiac events (nonfatal reinfarction or cardiac death)	D-dimer mean in men 508 ± 690 ng/mL vs women 564 ± 430 ng/mL	D-dimer had prognostic value in men (HR 2.35, 95%CI 1.27–4.35], *p* = 0.006) but not in women (HR 1.58, 95%CI 0.59–4.22, *p* = 0.360).
**ACS/CAD**									
Chen 2018 [39]	CAD (76.9% AMI)	238	64.4	21.1	P	24	All-cause mortality and CVEs (cardiac death and nonfatal outcomes: recurrent MI, TVR or re-admission due to advanced HF).	Mean D-dimer: 0.7 ± 1.1 mg/L.	OR for long-term CVEs: 1.526 (95% CI, 1.174–1.983), *p* = 0.002. D-dimer in multivariate Cox regression of CVEs: 1.420 (1.069–3.014), *p* = 0.046
Kosaki 2018 [40]	ACS (76.3% AMI)	400	71.1	27.2	P	27	CVEs (all-cause mortality, recurrent MI, unplanned repeat revascularization, surgical revascularization, fatal arrhythmia, admission for HF, and stroke.	Patients without CVEs: 1.67 mg/mL ±2.49 Patients with CVEs: 2.11 mg/mL ±2.72 *p* = 0.0003	Univariate analysis for D-dimer ≥0.84 mg/mL predicting CVEs: OR 2.49 (95%CI 1.54–4.11), *p* = 0.0001
ATLAS ACS-TIMI46 Trial Substudy 2018 [41]	ACS (73.9% AMI)	1834	Placebo 57.9 Rivaroxaban 57.2	23.2	Post-hoc RCT	6	Composite endpoint of CV death, myocardial infarction, or stroke	Baseline D-dimer (μg/mL) Placebo 0.39 (0.24–0.73) vs Rivaroxaban 0.42 (0.24–0.78) *p* = 0.370	Continuous D-dimer prognostic factor for composite outcome: univariate OR 1.15 (1.03–1.29) *p* = 0.015, multivariate OR 1.13 (1.0–1.28) *p* = 0.048
Mjelva, 2016 [42]	CAD (44.3% AMI)	871	69.5	38.7	P	84	All-cause mortality; a combined endpoint consisting of death or recurrent non-fatal MI; recurrent non-fatal MI alone.	194 (106–437) μg/L Median D-dimer in survivors vs non-survivors were for 153 vs 346 μg/L (*p* < 0.001)	D-dimer above 436 μg/L independently predicted mortality (4th vs 1st quartile HR 1.83 [95% CI 1.20–2.78], *p* = 0.005) Death or MI (4th vs 1st quartile HR 1.38 [95% CI 0.96–1.98], *p* = 0.08) Recurrent MI (4th vs 1st quartile HR 0.70 [95% CI 0.43–1.15], *p* = 0.16)
Gong 2016 [43]	CAD (29.2% AMI)	2209	58.58	25.9	P	18	Cardiac death, nonfatal MI, recurrence of MI, and stroke	Tertiles (μg/mL) 1: < 0.23, *n* = 816; 2: 0.23–0.36, *n* = 629; 3: > 0.36, *n* = 764.	D-dimer was linked to the severity of CAD (95% CI: 1.20–6.84, p = 0.005) Continuous D-dimer predictor of total outcome (HR = 1.22, 95% CI: 1.09–1.37, *p* = 0.001).
Charoensri 2011 [44]	ACS (61% AMI)	74	66	54.1	R	In-hospital	CHF, arrhythmias and death	D-dimer levels (μg/L) CHF: 1475 vs No CHF 385; Arrhythmia 5422 vs No arrhythmia 550; Death 5118 vs No death 2550	D-dimer levels correlated with complication of ACS (CHF; p < 0.001, arrhythmia; *p* = 0.007 and death; *p* = 0.009). D-dimer was significantly increased with the number of coronary arteries affected (*p* = 0.03).
Brugger-Andersen 2008 [45]	STEMI (15%), NSTEMI (29,3%), UA (9,4%), No ACS (46,3%)	871	69.6	39	P	24	All-cause mortality, CVEs (cardiac death or recurrent positive troponin T)	Quartiles of D-dimer (μg/l): Q1 < 106, Q2 ≥ 106–191, Q3 ≥ 191–438, Q4 ≥ 438.	In the univariate analysis highest D-dimer quartile predicted all-cause mortality compared with the lowest quartile (Q1) (OR 7.78 [95%IC, 3.95–15.33], p < 0.001), but not confirmed at multivariable logistic regression analysis (OR 1.80 [95%IC, 0.81 to 3.97]; *p* = 0.148).
Prisco 2001 [46]	CAD (52,9% AMI)	54	60 (44–75) 65 (38–81)	11.1	P	18	Restenosis	D-dimer before PCI: AMI (group 1) 55 ng/mL vs elective PCI (group 2) 29.0 ng/mL, *p* < 0.001	In group 1, D-dimer levels at the end of the procedure were higher in patients with restenosis than in those without (*p* < 0.005). Increased D-dimer in patients with restenosis (61%) than those without (25%, *p* < 0.05).
Oldgren 2001 [12]	ACS	320	66	–	P	29	Death, MI, and refractory angina during and after anticoagulant treatment in unstable CAD	< 82 μg/L (*N* = 105); 82–149 μg/L (*N* = 106); > 149 μg/L (*N* = 103)	No difference in clinical outcome at 72 h, 7 days and 30 days. During long-term follow-up, there was a relation between higher baseline levels of D-dimer and increased mortality (*p* = 0.003).

Study design: *A* ambispective, *P* prospective, *R* retrospective

§including recurrent MI, required renal replacement therapy, stent thrombosis, bleeding and length of hospital stay, hospital costs, and mortality and long-term CVEs (mortality, stent restenosis, non-fatal MI and TVR)

*ACS* acute coronary syndrome, *AMI* acute myocardial infarction, *AUC* area under curve, *CABG* coronary artery bypass grafting, *CAD* coronary artery disease, *CHF* congestive heart failure, *CI-AKI* Contrast-induced acute kidney injury, *CV* cardiovascular, CVEs cardiovascular events, *ESRD* end-stage renal disease, *HF* heart failure, *HFpEF* heart failure with preserved ejection fraction, *HFrEF* heart failure with reduced ejection fraction, *HR* hazard ratio, *NSTEMI* non-ST segment elevation myocardial infarction, *NT-proBNP* N-terminal pro-B-type natriuretic peptide, *OR* odds ratio, *PCI* percutaneous coronary intervention, *STEMI* ST segment elevation myocardial infarction, *TIA* transient ischemic attack, *TIMI* Thrombolysis in Myocardial Infarction, *TVR* target vessel revascularization, *UA* unstable angina

### Statistical analysis

When available, hazard ratios (HRs) and odds ratios (ORs) were recorded from each study.

A separate meta-analysis was conducted for each endpoint, using a hierarchical Bayesian model which therefore would take into account heterogeneity among studies. This technique was chosen due to the limited number of studies included. Formally, each study-specific effect was assumed to be distributed according to a log-Gaussian random variable, centered on a pooled effect. The variance on the log-scale was assumed to correspond to 1.25 times the squared standard error, as evaluated through confidence intervals reported in each study.

All analyses were conducted with R version 3.5.1, using the ‘metafor and ‘adaptMCMC’ packages.

## Results

### Characteristics of studies

The association between D-dimer levels and clinical outcomes has been investigated in populations enrolled using different definitions of ischemic heart disease, such as CAD, ST segment elevation myocardial (STEMI), non-ST segment elevation myocardial infarction (NSTEMI), acute MI and acute coronary syndrome (ACS). Characteristics of these studies have been separately described according to each definition (Table 1). Supplementary Table 2 and Supplementary Table 3 display the method used to dose D-dimer, cut-off levels and antithrombotic therapy across included studies.

#### Cardiovascular events

PRISMA flow diagram showing study search strategy about cardiovascular events is reported in Supplementary Fig. 1. After screening, 42 potentially eligible studies were identified and were considered for detailed analysis (Supplementary Fig. 1); 32 studies were included in the systematic review with 28,869 patients. Of these, 9 were retrospective, 20 prospective, 1 ambispective, 1 cross sectional study and 1 post-hoc analysis of a randomized clinical trial.

According to different inclusion criteria, 13 studies included STEMI, 3 NSTEMI, 7 MI, 9 CAD patients.

#### ST segment elevation myocardial (STEMI)

Overall, a sample of 7729 patients was analysed. The mean age ranged from 41 to 70.2 years and the proportion of women ranged from 8.45 to 54.7%. Four studies investigated outcomes occurred during hospital stay, while 7 studies investigated long-term outcomes (Table 1).

Overall, 11 studies showed an association between high plasma D-dimer levels and CVEs in STEMI patients. In particular, D-dimer predicted worse prognosis in all studies considering in-hospital outcomes, and in 5 out of 7 studies with long-term follow-up (Table 1).

#### Non-ST segment elevation myocardial infarction (NSTEMI)

A total of 1949 NSTEMI patients, with a mean age that ranged from 57.2 to 67.4 years was included. The proportion of female patients ranged from 24.8 to 35.8%. All studies investigated long-term prognosis. Overall, 1 out of 3 studies showed a positive association between high plasma D-dimer levels and CVEs incidence. However, one study showing no association between D-dimer and outcomes showed an association between D-dimer and NSTEMI severity, as assessed by Global Registry of Acute Coronary Events (GRACE) and thrombolysis in myocardial infarction (TIMI) risk scores, and included only mortality as endpoint; the other study had a short follow-up (6 months) and included also heart failure among cardiovascular outcomes (Table 1).

#### Myocardial infarction

Seven studies included a total of 12,320 patients with MI, with a mean age ranging from 62.8 to 74.2 years. The proportion of women ranged from 20 to 33.6% and 2 studies investigated in-hospital outcomes. Overall, 4 out of 7 studies consistently showed that D-dimer associated with CVEs (Table 1). Two studies also investigated the existence of a sex-based difference in the prognostic role of D-dimer providing conflicting evidence. Indeed, the study by Wang et al. found that D-dimer predicted all-cause mortality in women but not men. In this study, women were significantly older than men (74 vs 61 years). The opposite was found in the THROMBO study, in which however the mean age was lower and the endpoint included a composited of non-fatal events and cardiovascular death (Table 1).

#### Acute coronary syndrome (ACS) or CAD

Nine studies included 6871 ACS/CAD patients with a mean age ranging from 57.2 to 71.1 years. The proportion of female patients ranged from 11.1 to 54.1%. Overall, 8 out of 9 studies found that D-dimer was a predictive risk factor for CVEs in patients with ACS or CAD. In one study [45], the association between D-dimer and mortality was not confirmed at multivariable analysis; however, this study included a very heterogeneous population mixing STEMI, NSTEMI, unstable angina and no ACS patients.

#### Results of meta-analysis

**Fig. 1:**
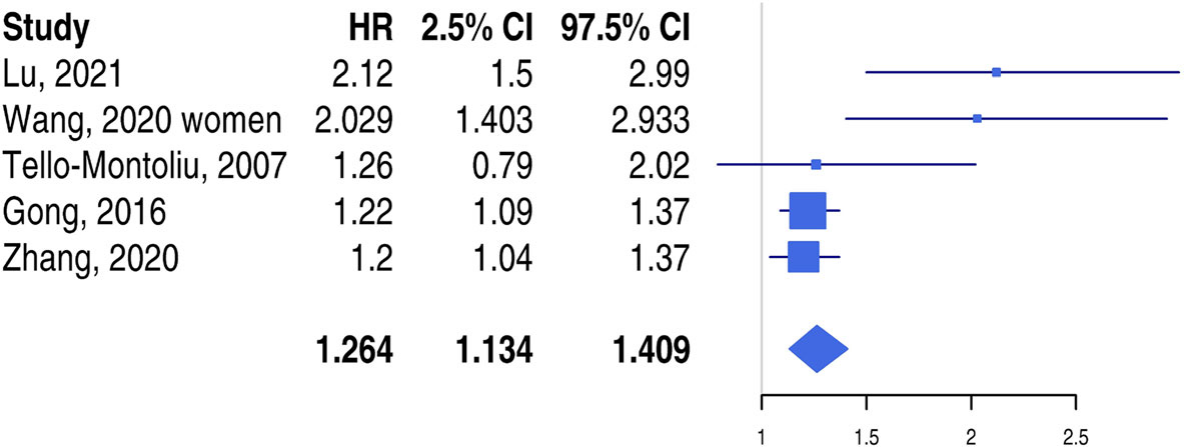
Forest plot of the hazard ratio for the risk of composite endpoints according to D-dimer values in patients with acute MI. HR: hazard ratio; CI: confidence interval

Five studies satisfied inclusion criteria and were included in the metanalysis: 4 prospective and 1 retrospective studies (Table 1) with a total of 8616 patients. Only studies reporting HR for continuous values were formally analysed. Median follow-up was 13.2 months with a total of 626 CVEs. The weighted mean age was 61.9 years. The percentage of women ranged from 25.9 to 100% (in one study). The pooled HR for D-dimer levels and CVEs was 1.264 (95% CI 1.134–1.409) (Fig. 1).

#### No-reflow phenomenon in STEMI patients

We also performed a systematic search using “D-dimer” and “no-reflow”. After screening, 7 potentially eligible studies were identified (3 prospective, 3 retrospective and 1 ambispective) (Supplementary Fig. 2). A total of 4195 STEMI patients were included, with mean age ranged from 52.6 to 64.0 mean years (female patients from 16 to 53.7%). Overall, 1327 patients (31.6%) had no-reflow phenomenon (Table 2). Five out of 7 studies showed a positive correlation of D-dimer levels with no-reflow phenomenon. Given the high variability in the methods of each study (cut-off of D-dimer used, measures of association) a formal analysis was not performed.

**Table 2:** Clinical characteristics of studies investigating no-reflow phenomenon included in the systematic review

Author/ year	N^**a**^	Age (years)	Women (%)	Study design	Events	D-dimer levels		Odds Ratio	Low CI	High CI	Main findings
						No-reflow group	Control group				
Gong 2020 [47]	229	63.7	17	R	28	1600 ± 1400 ng/mL	500 ± 600 ng/mL	2.520	1.160	5.470	D-dimer level can independently predict no-reflow after PCI. D-dimer value of 530 ng/mL was an effective cut-off point for postprocedural no-reflow with 85.7% of sensitivity and 67.7% of specificity (AUC = 0.78; *p* = 0.049).
Huang 2020 [18]	1165	63.5	17	R	165	≥ 800 ng/mL	< 800 ng/mL	1.399	0.929	2.106	D-dimer group had more frequently no-reflow (13.1% vs. 18.8%. *p* = 0.028).
Cheng 2019 [48]	218	58.7	17.5	R	39	410.3 ± 237.2 ng/mL	536.9 ± 291.7 ng/mL	1.001	1.000	1.003	No-reflow patients were older, diabetics, with longer pain-to balloon time, lower blood pressure, higher platelet count and higher levels of D-dimer and Cystatin C.
Zhang 2018 [22]	926	52.6	53.7	P	435	508.5 ± 254.7 ng/mL	272.0 ± 218.9 ng/mL	2.563	1.910	3.439	Multivariate OR for predicting no-reflow for D-dimer above mean (383.1 ng/mL).
Gao 2018 [23]	822	62.5	46.1	P	418	533.0 ± 244.0 ng/mL	323.4 ± 224.4 ng/mL	4.212	2.973	5.967	Diabetic patients with high D-dimer levels showed higher risk of no-reflow. Sensitivity of high plasma D-dimer levels in predicting no-reflow was 0.766.
Sarli 2015 [25]	266	64	38	P	63	686 ± 236 μg/l	418 ± 164 μg/l	1.005	1.003	1.007	D-dimer levels predicted no-reflow (OR: 1.005; 95% CI: 1.003–1.007; *p* < 0.001). Optimal cut-off for no-reflow was 549 μg/l.
Erkol 2014 [15]	569	56	16	A	179	720 (280–1490) mg/L	350 (170–620) mg/L	1.640	1.260	2.140	D-dimer (per each 1 mg/L increase) predictor of angiographic no-reflow (*p* < 0.001).

^a^all STEMI patients. Study design: *A* ambispective, *P* prospective, *R* retrospective. *PCI* percutaneous coronary intervention. *CVEs* cardiovascular ev

## Discussion

Our study shows that most studies including patients with ischemic heart disease showed a consistent association between D-dimer levels and worse clinical outcomes. This association was particularly evident in studies investigating in-hospital adverse events.

**Fig. 2:**
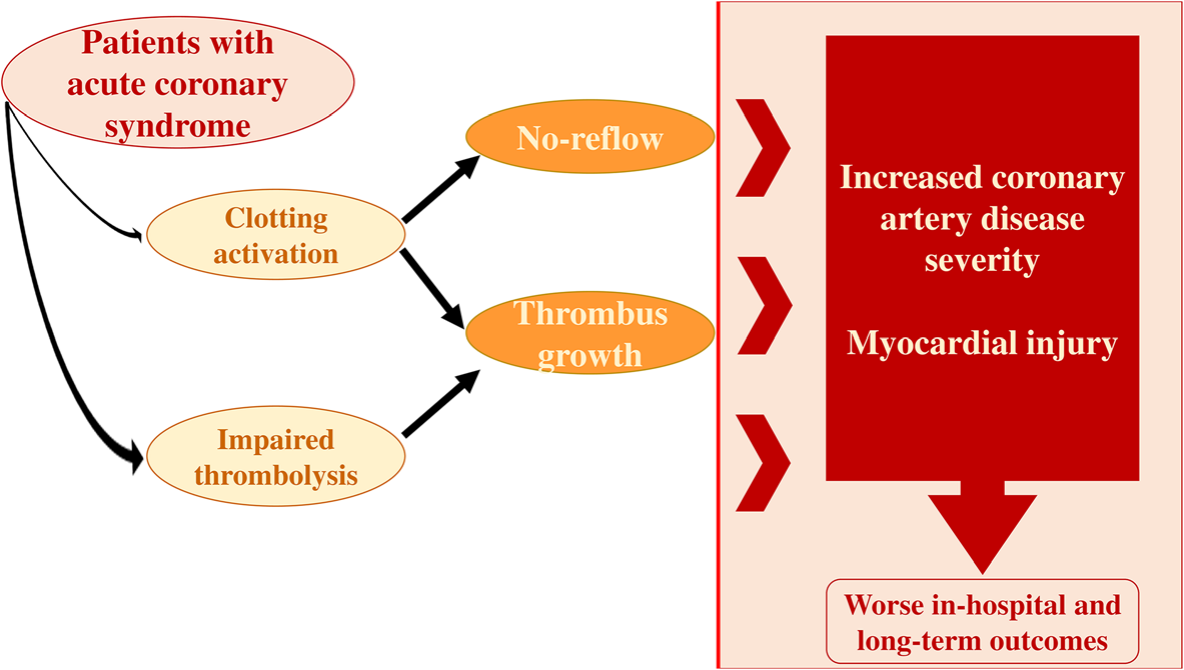
Pathophysiological mechanisms linking clotting activation and impairment of thrombolysis with the severity of acute coronary syndrome presentation and clinical outcomes

The rationale of investigating D-dimer levels in CAD patients relies on the persistent hypercoagulable state described in MI patients, which may lead to worse clinical outcomes in these patients through several mechanisms [14]. Clotting activation may contribute to thrombus growth and no-reflow phenomenon, along with impaired thrombolysis which has been frequently described in CAD patients [49], all factors contributing to an increased CAD severity and myocardial injury **(**Fig. 2**).**

Reperfusion fails to occur after thrombolytic therapy in approximate 40% of patients, as shown by a study including 427 STEMI patients undergo rescue PCI [50]. Indeed, high D-dimer levels have been shown to be significantly higher in lysis-resistant thrombi from STEMI patients [51].

The pathogenesis of no-reflow remains unclear, but it is likely to be multifactorial, including endothelial damage platelet and clotting activation leading to thrombus formation at the level of small vessels [52]. Few studies investigated the occurrence of no-reflow in STEMI patients undergoing PCI and its relationship with D-dimer [15, 22, 23, 25, 47, 48]. In 229 consecutive STEMI patients, X. Gong et al. [47] found that a D-dimer value of 530 ng/mL (FEU) was associated with postprocedural no-reflow, with 85.7% of sensitivity and 67.7% of specificity (area under the curve [AUC] = 0.78; *p* = 0.049). Similarly, Sarli et al. found that optimal D-dimer cut-off value for predicting no-reflow was 549 μg/l (FEU) [25]. In 218 STEMI patients, Cheng et al. [48] found that no-reflow patients were older, diabetics, with longer pain-to balloon time, lower blood pressure, higher platelet counts and higher levels of D-dimer and Cystatin C than patients without no-reflow.

### Risk stratification using D-dimer

In the past decades, an increasing number of biomarkers have been tested to improve cardiovascular risk stratification in patients with ACS [6, 53]. In particular, some studies suggested that the use of a combination of multiple biomarkers may improve risk stratification. This is the case of D-dimer, which if used alone is currently recommended to only exclude acute venous thromboembolism, but its addition to other biomarkers/risk scores gave promising results.

Indeed, the addition of D-dimer has been investigated to some biomarkers such as C-reactive protein (CRP), NT-proBNP and clinical scores, such as the Global Registry of Acute Coronary Events (GRACE) risk score has been investigated. Fu et al. included D-dimer levels ≥2.4 mg/L FEU in a risk score model together with CRP, left ventricular ejection fraction, age ≥ 65 years old and heart rate [32]. In ROC curve analysis, this model demonstrated a good power in predicting in-hospital mortality (AUC = 0.895, 95% CI 0.814–0.96; *p* <  0.001), better than the predictive power of the GRACE risk score alone (AUC = 0.754, 95% CI 0.641–0.868; p <  0.001). D-dimer has been tested combined to GRACE score also in the study by Lin et al. who found that the combination of NT-pro-BNP and D-dimer improved the predictive accuracy of GRACE score for all-cause death [29]. In 5923 ACS patients undergoing PCI, GRACE score combined with D-dimer achieved a better prognostic performance than GRACE score, and D-dimer could significantly improve the prognostic performance of GRACE score [35]. This result is in keeping with that described by Yu et al., in which [35] D-dimer levels significantly improved the prognostic performance of GRACE score.

### Management and therapeutic implications

Altogether, current evidence suggest that D-dimer may identify patients at higher risk for a more severe CAD and worse prognosis. Of note, clotting activation is not lowered by antiplatelet therapy [54], as shown by previous studies reporting no effect of aspirin and DAPT in reducing D-dimer levels [55]. Conversely, oral and parenteral anticoagulation, in addition or not to DAPT, has been demonstrated to decrease D-dimer levels in patients with MI [41, 56, 57]. Thus, ATLAS ACS 2-TIMI 51 and COMPASS trials have recently proved the utility of adding low-dose anticoagulation to antiplatelet medications. In ATLAS ACS 2-TIMI 51, introduction of low-dose rivaroxaban therapy (2.5 mg or 5 mg twice daily, with 93% of patients on DAPT) has shown to be effective in reducing the risk of death from cardiovascular causes, myocardial infarction, or stroke [58], even if the use of rivaroxaban increased the risk of major bleeding and intracranial haemorrhages. Similarly, the COMPASS trial demonstrated that rivaroxaban plus aspirin was effective in decreasing the primary outcome of cardiovascular death, stroke or MI with a 22% reduction in the net clinical benefit endpoint (defined as the composite of primary outcome, fatal bleeding and symptomatic bleeding into a critical organ/area) [59]. Moreover, a sub-analysis of the COMPASS trial has shown that patients with high thrombotic profile (i.e., peripheral arterial disease) may benefit more from adding anticoagulation therapy to reduce residual thrombotic risk.

However, these studies did not stratified patients according to D-dimer levels. Data from the literature let to hypothesize that the benefit conferred by an association therapy of oral anticoagulation and antiplatelet therapy may be even more evident in patients with the features of clotting activation (i.e. high D-dimer). This aspect would deserve a specific investigation.

Another group of patients who deserve particular attention is represented by patients presenting with no-reflow. Preliminary data from the literature suggest an association between high-D-dimer levels and no-reflow. Moreover, in MI patients who cannot undergo cardiac catheterization, D-dimer concentrations may also be helpful to predict severity of coronary disease [60].

**Fig. 3:**
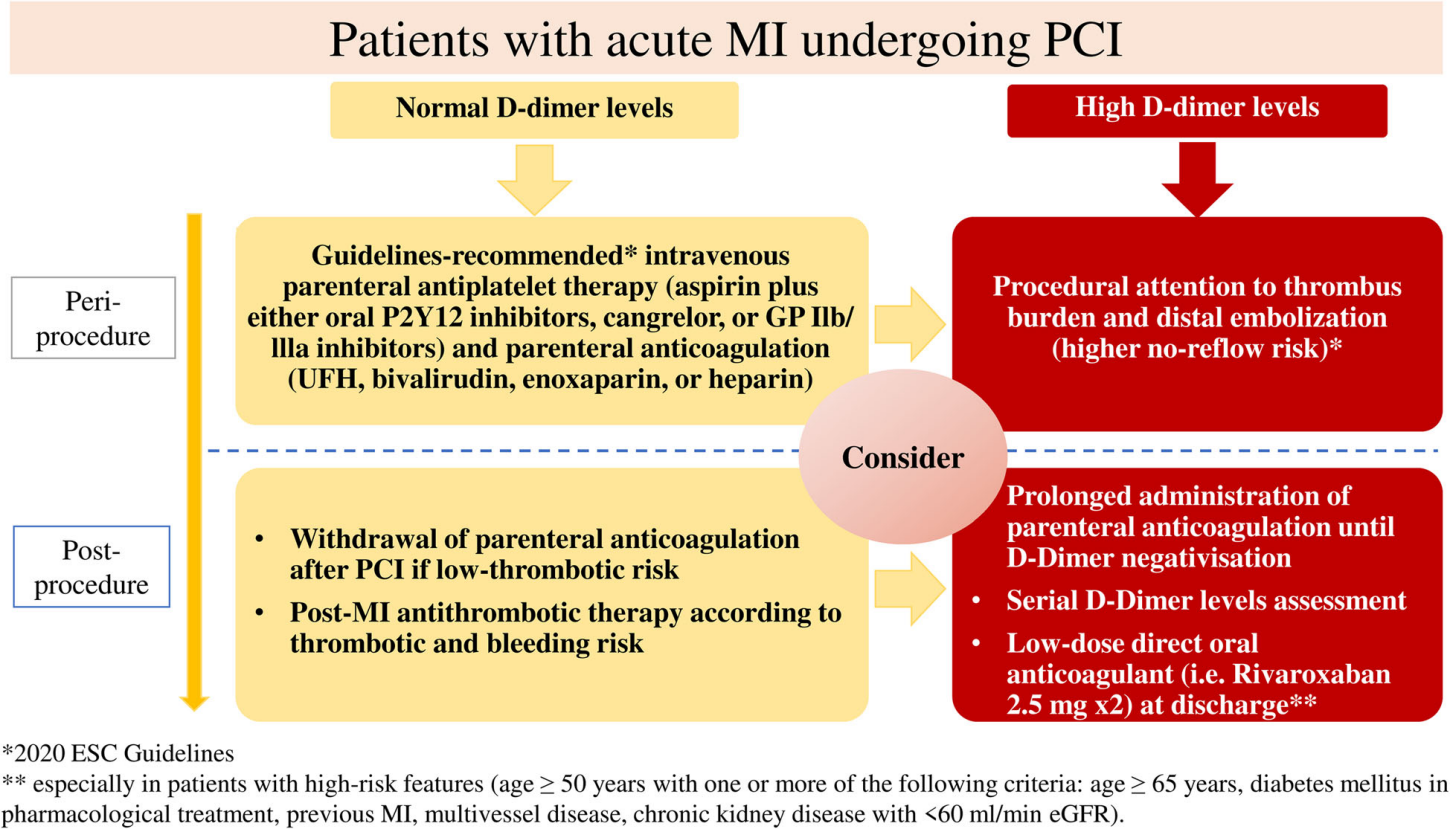
Proposed flow-chart for the management of patients with acute MI according to D-dimer values. MI: myocardial infarction; PCI: percutaneous coronary intervention; UFH: unfractionated Heparin

Finally, D-dimer levels might be helpful in identifying MI patients at high risk of complications and worse outcome. In Fig. 3, we propose a D-dimer guided strategy to tailored therapy in patients at higher thrombotic risk such as those with MI undergoing PCI. Firstly, MI patients with high D-dimer levels may benefit from specific pharmacological management and technical procedures to reduce the probability of no-reflow phenomenon. These include the use of glycoprotein IIb/IIIa inhibitors, higher heparin dose and direct stenting without predilation [61–64]. Those high-risk patients might also take advantage from prolonged parenteral anticoagulation after PCI during in-hospital staying to reduce early complications. At discharge, patients presenting with high-risk features, including presence of comorbidities, multivessel disease, age > 65 years, recurrent MI, multiple stent insertion, may benefit from adding a low dose of oral anticoagulant agent to conventional antiplatelet therapy to reduce the residual thrombotic risk (i.e., rivaroxaban 2.5 mg twice daily).

Study limitations. A formal analysis for D-dimer against no-reflow phenomenon was not performed given the high variability in the methods of each study (cut-off of D-dimer used, measures of association) was not performed.

## Conclusions

In conclusion, given its association with CVEs, the use of D-dimer may help physicians to improve outcome and manage residual thrombotic risk in MI patients. D-dimer may therefore represent a valuable guide in the difficult decision-making process on the use of oral anticoagulation in addition to currently recommended antithrombotic therapy in patients with ACS.

## Supplementary Information

**Additional file 1.** Supplementary data.

## Data Availability

The datasets used and/or analysed during the current study are available from the corresponding author on reasonable request.

## Abbreviations

CAD: Coronary artery disease
MI: Myocardial infarction
PCI: Percutaneous coronary intervention
CVEs: Cardiovascular events
STEMI: ST segment elevation myocardial
NSTEMI: Non-ST segment elevation myocardial infarction
ACS: Acute coronary syndrome
CRP: C-reactive protein
GRACE: Global registry of acute coronary events
TIMI: Thrombolysis in myocardial infarction
