# Correction to: D-dimer for risk stratification and antithrombotic treatment management in acute coronary syndrome patients: a systematic review and metanalysis

**DOI:** 10.1186/s12959-022-00366-2

**Published:** 2022-02-09

**Authors:** Flavio Giuseppe Biccirè, Alessio Farcomeni, Carlo Gaudio, Pasquale Pignatelli, Gaetano Tanzilli, Daniele Pastori

**Affiliations:** 1 Department of Clinical Internal, Anesthesiological, and Cardiovascular Sciences, Sapienza University of Rome, Rome, Italy; 2 Department of General and Specialized Surgery “Paride Stefanini,” Sapienza University of Rome, Rome, Italy; 3 Department of Economics and Finance University of Rome “Tor Vergata”, Rome, Italy

**Correction to:** **Thrombosis J** **19, 102 (2021).**

**https://doi.org/10.1186/s12959-021-00354-y**

Following publication of the original article [1], it was reported that there was an error in the author affiliations. The details of affiliation 2 and 3 were transposed.

The correct affiliations are given in this Correction article and the original article [1] has been updated.
