# Supplementary material for: D-dimer for risk stratification and antithrombotic treatment management in acute coronary syndrome patients: a systematic review and metanalysis
Source: Thromb J. 2021 Dec 18;19:102. doi: 10.1186/s12959-021-00354-y (PMC8684263; doi:10.1186/s12959-021-00354-y)
Supplement: Supplementary file 1 — Additional file 1. Supplementary data. [file 12959_2021_354_MOESM1_ESM.docx]

**SUPPLEMENTARY DATA**

**Supplementary Figure 1. PRISMA flow diagram of study research investigating composite of major adverse cardiac events (MACE), cardiovascular death and all-cause mortality.**

**
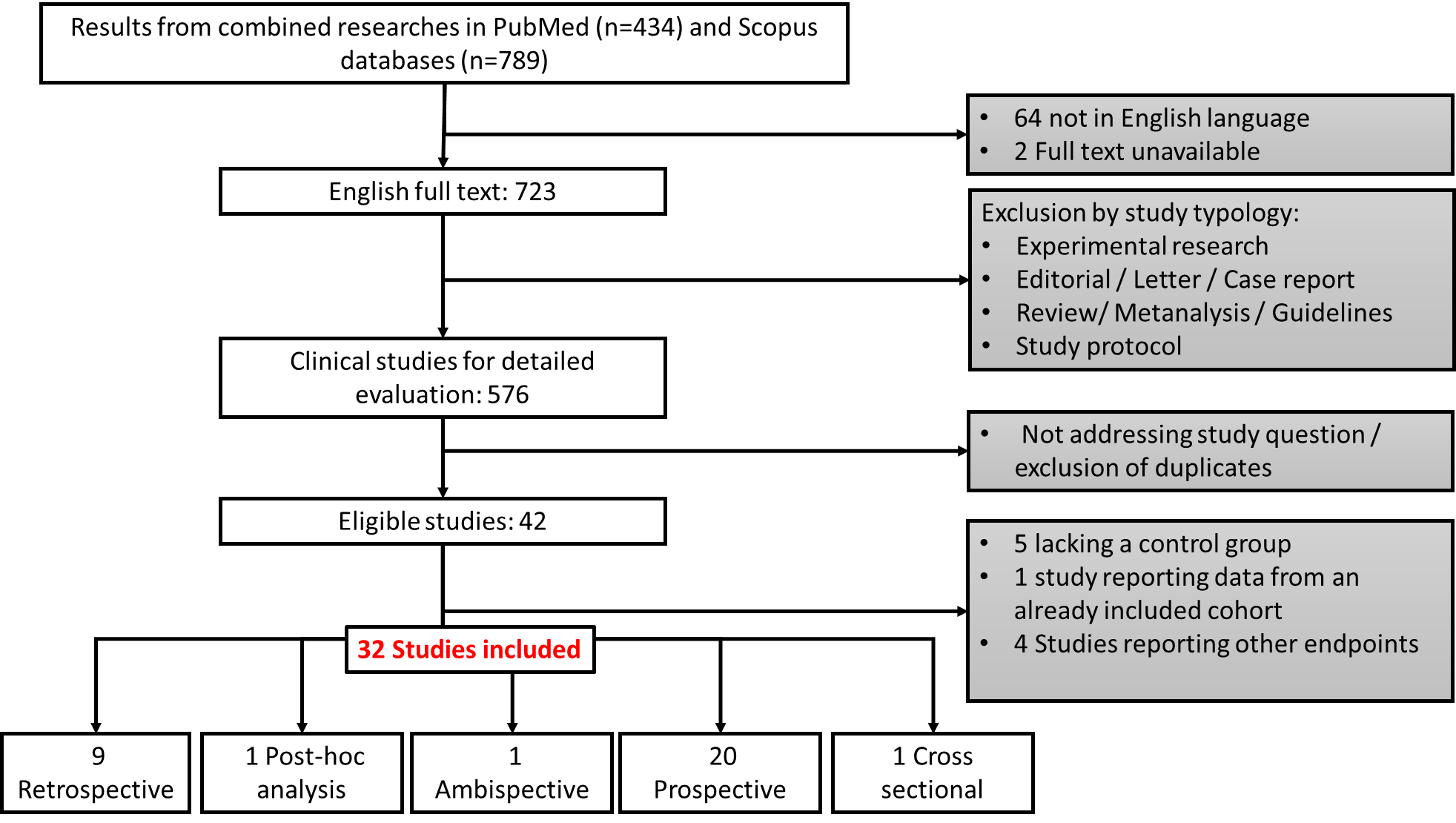
**

**Supplementary Figure 2. PRISMA flow diagram of study research investigating no-reflow phenomenon.**

**
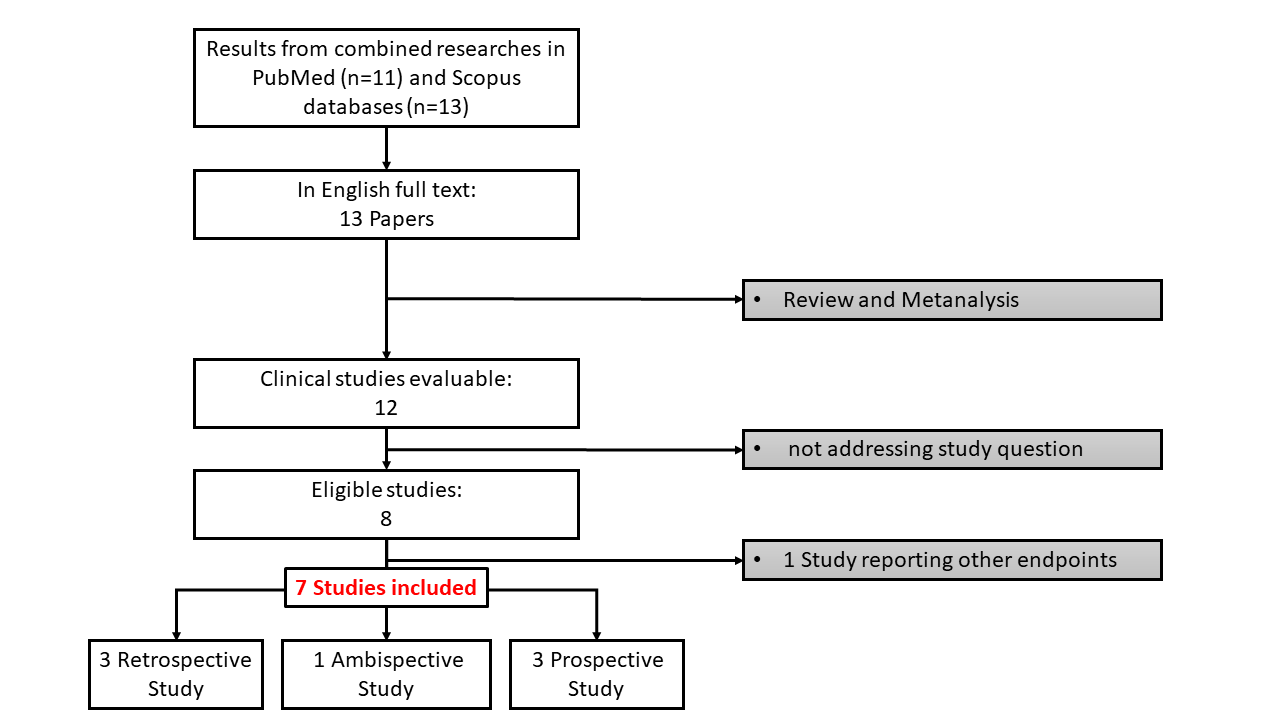
**

**Supplementary Table 1. The Newcastle–Ottawa scale score for each study.**

| *N°* | *Study* | *Selection* | *Comparability* | *Outcome* | *Total* |
| --- | --- | --- | --- | --- | --- |
| 1 | Gong 2020[61] | 4 | 2 | 3 | 9 |
| 2 | Lu 2021[28] | 4 | 2 | 1 | 7 |
| 3 | Tello-montoliu 2007[52] | 4 | 0 | 1 | 5 |
| 4 | Zhang 2018[21] | 4 | 2 | 2 | 8 |
| 5 | P. Wang 2020[53] | 4 | 0 | 1 | 5 |

**Supplementary Table 2. D-dimer investigation features and antithrombotic therapy of studies investigating major adverse cardiac events and all-cause mortality included in the systematic review.**

| **Author/ year** | **Setting** | **FEU/ DDU** | **Cut-off** | **D-Dimer method used** | **Systemic D-dimer collection** | **Antiplatelet therapy** | **Parenteral anticoagulation** | **Oral anticoagulation** |
| --- | --- | --- | --- | --- | --- | --- | --- | --- |
| Biccirè 2021[42] | STEMI | FEU | NR | NR | At admission | NR | NR | NR |
| Huang  2020 [43] | STEMI | NR | <0.8 mg/L | Immunoturbidimetry  method | Upon admission  (within 4 h). | All patients received a loading dose of aspirin (300 mg)  and P2Y12 antagonist (300 mg clopidogrel or 180 mg ticagrelor).  53.8% Tirofiban before stent | 2500 IU heparin administered  after successful puncture, and a weight dependent dose (up to  100 IU/kg) added for PCI. | NR |
| Luo  2020 [44] | STEMI | FEU | NR | Enzyme immunoassay using INNOVANCE  D-dimer (SIEMENS) | NR | All patients were given aspirin (300 mg), ticagrelor (180 mg),  or clopidogrel (300 mg) before the operation | NR | NR |
| Qi Zhou 2020 [45] | STEMI | NR | - CS200i/CS5100  automatic analyzer (Sysmex, Japan) with the normal D-dimer  value set between 0 and 0.55 μg/mL  - CA92121 analyzer  (San Diego, CA) with the normal value set at 0 to 600 ng/mL. | D-dimer levels were measured using an immunoturbidimetric test  (STA-Liatest D-dimer).  To unify the 2 sets of data, the D-dimer levels were instead by increased multiple, which was defined as the levels of D-dimer  divided by the upper limit of the normal range of D-dimer. | Immediately after admission | All patients were administered loading doses of antiplatelet  medications (300 mg aspirin and 180 mg ticagrelor) before coronary angiography | 3000 IU heparin was administered when the coronary anatomy was first defined | NR |
| Lin  2020 [46] | STEMI | FEU | NR | NR | At admission  before PCI | NR | NR | NR |
| Zhang  2018 [21] | STEMI | NR | NR | Human D-dimer ELISA kit  (EHDDIMER, Thermo Fisher Scientific, Waltham, MA, USA) with  a sensitivity of 0.08 pg/mL. | Before the PCI procedure  on the day of patient admission to the hospital | NR | bolus of heparin (5000 IU) administered  before the procedure | NR |
| Gao  2018 [22] | STEMI with T2DM | NR | NR | ELISA kit  (ab196269, Abcam, Cambridge, MA, USA) with a sensitivity  of 71 pg/mL. | At admission | NR | bolus of 5000 IU of heparin | NR |
| C. H. Hansen 2018 [47] | STEMI | NR | NR | Enzyme-linked immunosorbent assay (Asserachrom  D-dimer; Stago Diagnostica, Ansiere, France and Enzygnost  F1þ2; Siemens, Marburg, Germany, respectively). | Blood samples collected at a median time of 24 hours after onset of symptoms,  18 hours after PCI procedure. | 222 (23%) aspirin as home therapy | All patients received heparin during the PCI  procedure approximately 16 to 20 hours before blood sampling. | Patients on  oral anticoagulation were excluded from the study |
| B. Sarli 2015 [25] | STEMI | FEU | NR | MDA immunoturbidimetric assay; Organon Teknika | In the emergency department | Blood samples for D-dimer analysis obtained before administration of antithrombotic treatment | Blood samples for D-dimer analysis obtained before administration of antithrombotic treatment | Patients on  oral anticoagulation were excluded from the study |
| Ayhan Erkol 2014 [15] | STEMI | FEU | NR | Immunoturbidimetric  test (STA-Liatest D-dimer) on the STAR Evolution analyser  (Diagnostica Stago, Asnie‘res, France). | On admission | All patients received a 300 mg chewable aspirin and a  600 mg loading dose of clopidogrel on admission.  The use of GPI  (tirofiban)  was left to the primary operator’s discretion. | 70 U/kg intravenous standard heparin before the procedure. | NR |
| HORIZONS-AMI substudy 2014 [48] | STEMI | NR | NR | Alere Inc.,  San Diego, CA, using Luminex immunoassay methods | At study enrollment (before PCI procedure) | 1:1 ratio to treatment with  bivalirudin alone or with UFH plus a GPI | 1:1 ratio to treatment with  bivalirudin alone or with UFH plus a GPI | NR |
| Ozgur Akgul  2013 [49] | STEMI | FEU | NR | Chemiluminescence  enzyme immunoassay (CLEIA) method with the  Pathfast (Mitsubishi Chemical Medience Corporation,  (Tokyo, Japan) | On admission before  catheterization procedures | 300mg aspirin and  clopidogrel (600mg loading dosage) before coronary  angiography. | Heparin (100 IU/kg) administered when the coronary  anatomy was first defined. | NR |
| Javier Pineda 2010 [50] | AMI STEMI (85.9%) | NR | NR | ELISA techniques (Diagnostica STAGO, Paris, France) | Blood sampling was performed at least  3 months after the acute AMI and in the stable phase of the disease | NR | NR | NR |
| Lu  2021 [28] | NSTEMI | NR | NR | Roche Diagnostics (Mannheim, Germany) | Blood was collected within the first 12 hours after admission | Dual antiplatelet therapy in 1335 patients (98.4%) | Parel anticoagulants in 1334 patients (98.3%) | NR |
| Hulusi Satilmisoglu 2017 [51] | NSTEMI | NR | <500 ng/mL. | Immunoturbidimetric methods using commercial kits (Roche Diagnostics, Mannheim, Germany) with the aid of a Roche Cobas 6000 c501 analyzer (Roche Diagnostics) | At the time of hospitalization | NR | NR | NR |
| A. Tello-Montoliu 2007 [52] | NSTEMI | DDU | NR | ELISA (Dade Behring®, Brea, CA,  USA) | In the first 48 h after admission | Home therapy: 42.1% of patients on aspirin and 10.9% of patients on clopidogrel  In hospital: standard management as recommended  for ACS, with regard to aspirin, clopidogrel and GPI | In hospital: standard management as recommended  for ACS, with regard to LMWH | NR |
| Fu 2020 [27] | AMI with ESRD | FEU | NR | NR | Collected from  fasting patients on the first day after admission, | NR | Anti-coagulation  was generally performed using LMWH | NR |
| P. Wang 2020 [53] | AMI | FEU | NR | NR | At admission or in the following  morning | home therapy:  99.5% antiplatelet therapy | NR | home therapy:  44.2% on oral anticoagulants |
| Zhang  2020 [54] | AMI | NR | NR | NR | On initial presentation to  hospital and prior to administration of anticoagulant or antiplatelet use and PCI | In hospital: aspirin in 97% patients and P2Y inhibitors in 97.2% | NR | NR |
| Yu  2019 [29] | AMI | NR | The reference interval of D-dimer was 0–  252 ng/mL. | Latex agglutination assays  by an automatic coagulation analyzer (ACL TOP, BECKMAN  COULTER, USA) | On admission | NR | Exclusion criteria: samples collected  within 5 h after use of UFH or 12 h  after use of LMWH | Exclusion criteria: use of oral anticoagulants |
| REBUS study  2017 [55] | AMI | FEU | Reference interval was <500 µg/L | Enzyme  immunoassay (Asserachrome, Stago, France). | At inclusion in the study 3–5 days after index AMI  and at early follow-up 2–3 weeks after the index event | At discharge, >95% of patients had been prescribed dual  antiplatelet treatment | NR | At discharge, 6.4% of patients had been prescribed  oral anticoagulant treatment |
| M. SMID 2011 [56] | AMI | DDU | NR | D-dimer Plus test  (Dade Behring Inc., Liederbach, Germany) | In the Emergency Room (day  0; before administration of low-molecular-weight heparin) at day 4 (while on antithrombotic treatment) and  after 3 and 6 months | NR | NR | Use of oral anticoagulants was considered as an exclusion criteria. |
| THROMBO study  2000 [57] | AMI | DDU | NR | Enzyme-linked immunosorbent assay technique  (Dimertest, American Diagnostica, Greenwich, Connecticut) | Collected 2  months after AMI | 81.7% on aspirin at enrolment | NR | 17.8% on oral anticoagulants at enrollment |
| Chen, 2018 [58] | CAD (76.9% AMI) | ND | NR | Cobas 8000 biochemical autoanalyzer (Roche,  Tokyo, Japan). | At admission | NR | NR | NR |
| Kosaki 2018 [59] | ACS (76.3% AMI) | NR | NR | latex agglutination method (BML, Tokyo, Japan) | Serum biomarkers were obtained from the arterial sheath within  1 h after interventions. | 72 patients were taking dual antiplatelet therapy and  328 patients had been administered antiplatelet before the PCI | PCI under intravenous administration with heparin (3000–  10,000 units) aiming at an activated clotting time of 250 s. | NR |
| ATLAS ACS-TIMI46 Trial Substudy 2018 [34] | ACS (73.9% AMI) | FEU | NR | STA-Liatest D-di immune-turbidimetric assay | D-dimer levels were collected for a subset of subjects before the first dose, 3 to 24 hours after first dose, and on study day 30 and 180 | In-hospital  26.6% aspirin  73.3% aspirin + clopidogrel | NR | NR |
| O. R. Mjelva, 2016 [60] | CAD (44.3% AMI) | NR | and the lowest above  zero standard concentrations of the test were 100 µg/L | Immunoturbidimetric method on a coagulation  analyser with reagents from Biopool (Umea, Sweden) | Immediately following  admission | Treatment prior to admission:  Aspirin 38.6% of patients  Clopidogrel 1.8% of patients | Blood samples drawn before treatment with heparin | NR |
| P. Gong 2016 [61] | CAD (29.2% AMI) | FEU | The normal reference range of plasma D-dimer was < 0.5 µg/mL | Immunoassays turbidimetry  (STA Compact Diagnostica Stago assay instrument, France) | At 7:00 AM in the next morning of the day of admission | In-hospital use of heparin not reported | NR | Patients with anticoagulant therapy before admission were excluded |
| Charoensri 2011 [62] | ACS (61% AMI) | FEU | Range of detected plasma  D-dimer was 45-10000 µg/L FEU | Enzyme immunoassay and  enzyme linked fluorescent assay (ELFA) technique.  Using the commercial kits and assay of Vidas D-dimer  ExclusionTM(DD2) (BioM’erieux, Lyon,USA) | Measured as quick as  when disease was confirmed and invasive strategy was  chosen. | NR | NR | Patients with anticoagulant therapy before admission were excluded |
| Trygve Brugger-Andersen 2008 [17] | STEMI (15%), NSTEMI (29,3%), UA (9,4%), No ACS (46,3%) | NR | The lowest above zero  standard concentrations for the test were 100 µg/l. | Coagulation analyzer  with assay reagents from Biopool (Umea,Sweden) | Immediately following admission | Treatment prior to admission:  Clopidogrel 1.8% of patients  Aspirin 62.3% of patients | NR | NR |
| D. Prisco 2001 [63] | CAD (52,9% AMI) | NR | Control value <60 ng/mL | NR | Before and immediately after (within 60 min) PCI | NR | NR | NR |
| J. Oldgren 2001 [12] | ACS | NR | Reference range: 10-130 µg/L | ELISA (TintElize D-dimer, Biopool) | At baseline before the start of treatment | Aspirin was given to 96% of the patients within the first day but ticlopidine  was not allowed. | Study protocol: Patients with unstable coronary artery disease were randomized to a 72-hour infusion with either inogatran, a low-molecular-mass direct thrombin inhibitor, or UFH | Oral anticoagulants were not allowed |

**ACS: acute coronary syndrome; AMI: acute myocardial infarction; CAD: coronary artery disease; DDU: D-Dimer units; FEU: fibrinogen equivalent units; GPI: glycoprotein IIb/IIIa inhibitor; LMWH: low molecular weight heparin; NR: not reported; NSTEMI: non-ST segment elevation myocardial infarction; PCI: coronary artery intervention; ; STEMI: ST segment elevation myocardial infarction; UA: unstable angina; UFH: unfractionated heparin.**

**Supplementary Table 3. D-dimer investigation features and antithrombotic therapy of studies investigating no-reflow phenomenon included in the systematic review.**

| **Author/ year** | **Setting** | **FEU/ DDU** | **Cut-off** | **D-Dimer method used** | **Systemic D-dimer collection** | **Antiplatelet therapy** | **Parenteral anticoagulation** | **Oral anticoagulation** |
| --- | --- | --- | --- | --- | --- | --- | --- | --- |
| X. Gong  2020 [23] | STEMI | FEU | NR | MDA  immunoturbidimetric assay; Sysmex, CA7000 | on admission in the emergency department | All patients received a 300 mg chewable aspirin and a 300-mg loading dose of clopidogrel or a 180-mg loading dose of ticagrelor on admission | all patients received a 100 U/kg intravenous standard heparin | NR |
| Huang  2020 [43] | STEMI | NR | <0.8 mg/L | Immunoturbidimetry  method | upon admission (within 4 h). | All patients received a loading dose of aspirin (300 mg) and P2Y12 antagonist (300 mg clopidogrel or 180 mg ticagrelor) upon  the diagnosis of STEMI.  53.8% of patients with Tirofiban before stent | A total of 2500 IU heparin  and a weight dependent dose (up to 100 IU/kg) added for PCI. | NR |
| Cheng  2019 [24] | STEMI | NR | NR | NR | At admission | All patients received 300 mg aspirin, 600 mg clopidogrel. The GPI tirofiban  was administered during the primary PCI according to the operator’s preference | standard heparin (70 U/kg of body weight) before coronary artery angiography/PCI | NR |
| H. Zhang  2018 [21] | STEMI | NR | NR | Human D-dimer ELISA kit  (EHDDIMER, Thermo Fisher Scientific, Waltham, MA, USA) with  a sensitivity of 0.08 pg/mL | venous blood before the PCI procedure  on the day of patient admission to the hospital | NR | A bolus of heparin (5000 IU) administered  before the procedure | NR |
| Gao  2018 [22] | STEMI with T2DM | NR | NR | ELISA kit  (ab196269, Abcam, Cambridge, MA, USA) with a sensitivity  of 71 pg/mL | upon admission | NR | bolus of 5000 IU of heparin | NR |
| Sarli  2015 [25] | STEMI | FEU | NR | MDA immunoturbidimetric assay; Organon Teknika | during the initial evaluation of patients in the emergency department | Blood samples for D-dimer analysis obtained before administration of antithrombotic treatment | Blood samples for D-dimer analysis obtained before administration of antithrombotic treatment | Patients on  oral anticoagulation were excluded from the study |
| Erkol  2014 [15] | STEMI | FEU | NR | Immunoturbidimetric  test (STA-Liatest D-dimer) on the STAR Evolution analyser  (Diagnostica Stago, Asnie‘res, France) | on admission | All patients received a 300 mg chewable aspirin and a 600 mg loading dose of clopidogrel on admission.  The use of GPI  (tirofiban)  was left to the primary operator’s discretion | 70 U/kg intravenous standard heparin before the procedure | NR |

**FEU: fibrinogen equivalent units; GPI: glycoprotein IIb/IIIa inhibitor; NR: not reported; PCI: coronary artery intervention; STEMI: ST segment elevation myocardial infarction; T2DM: type 2 diabetes mellitus.**
